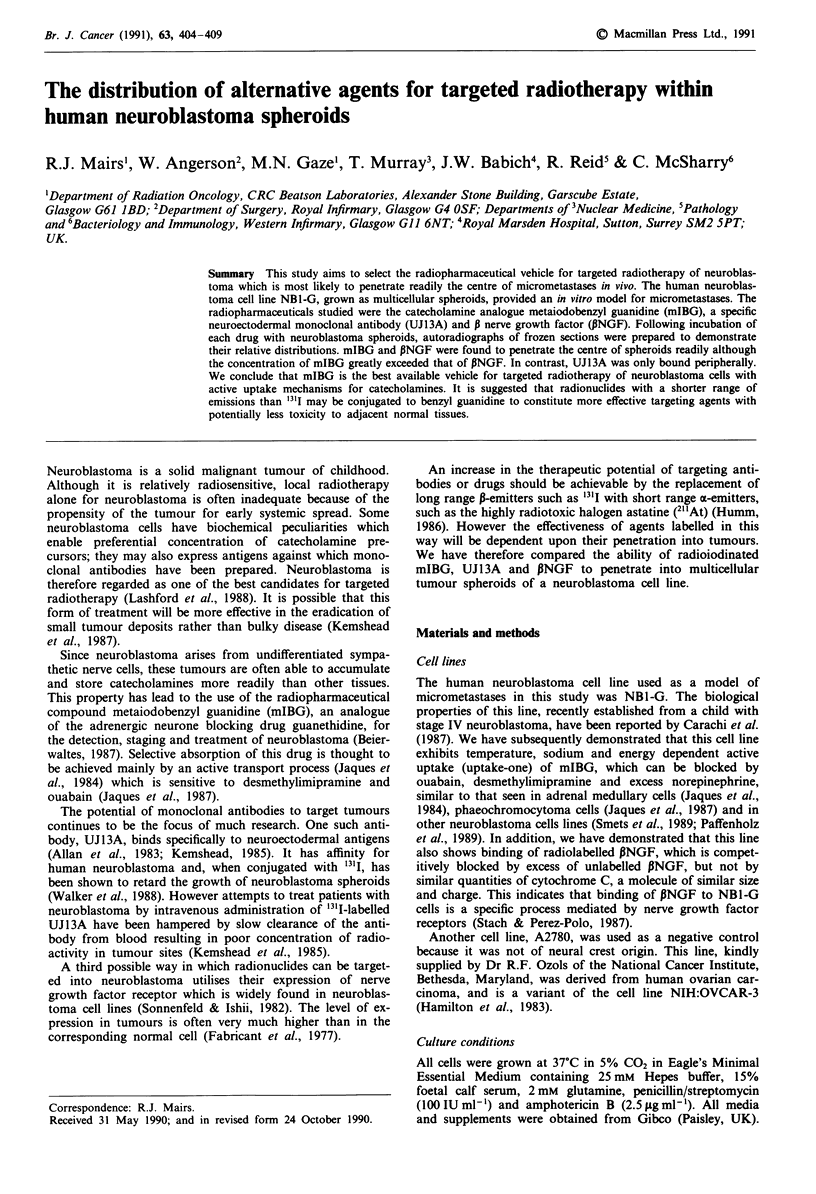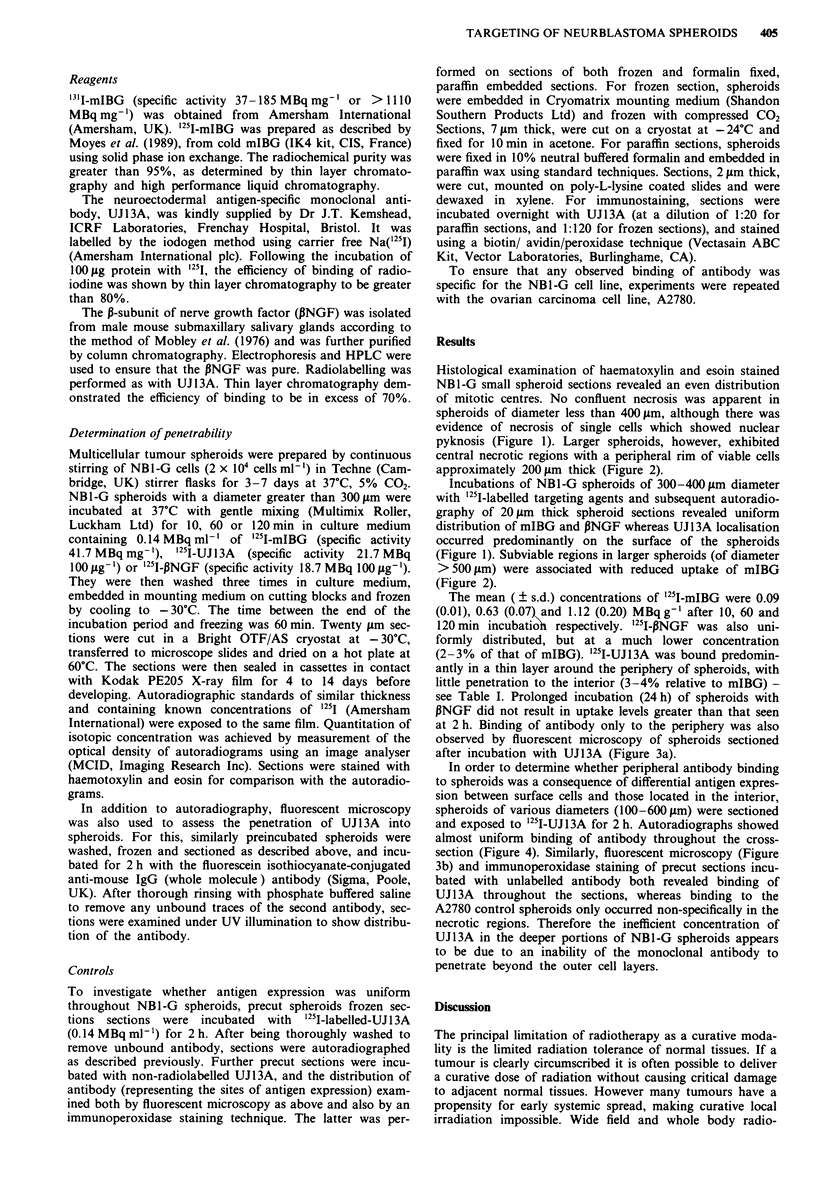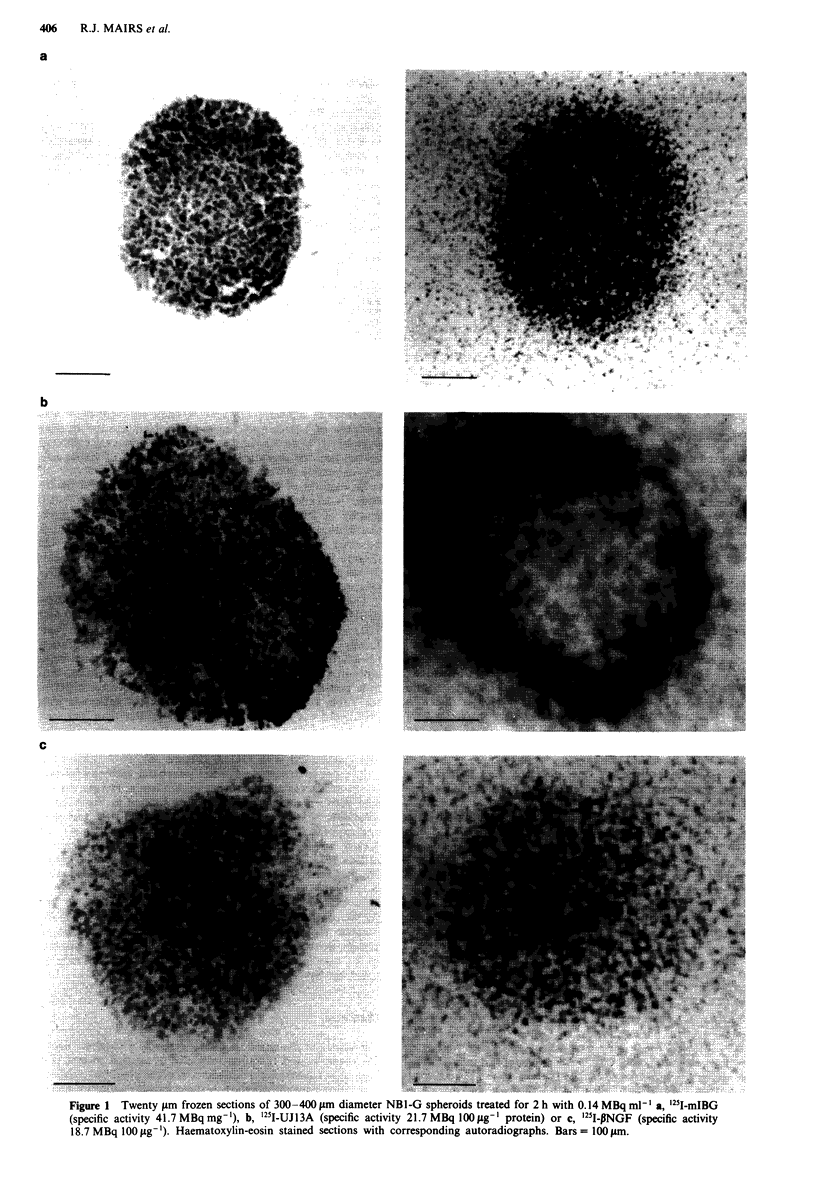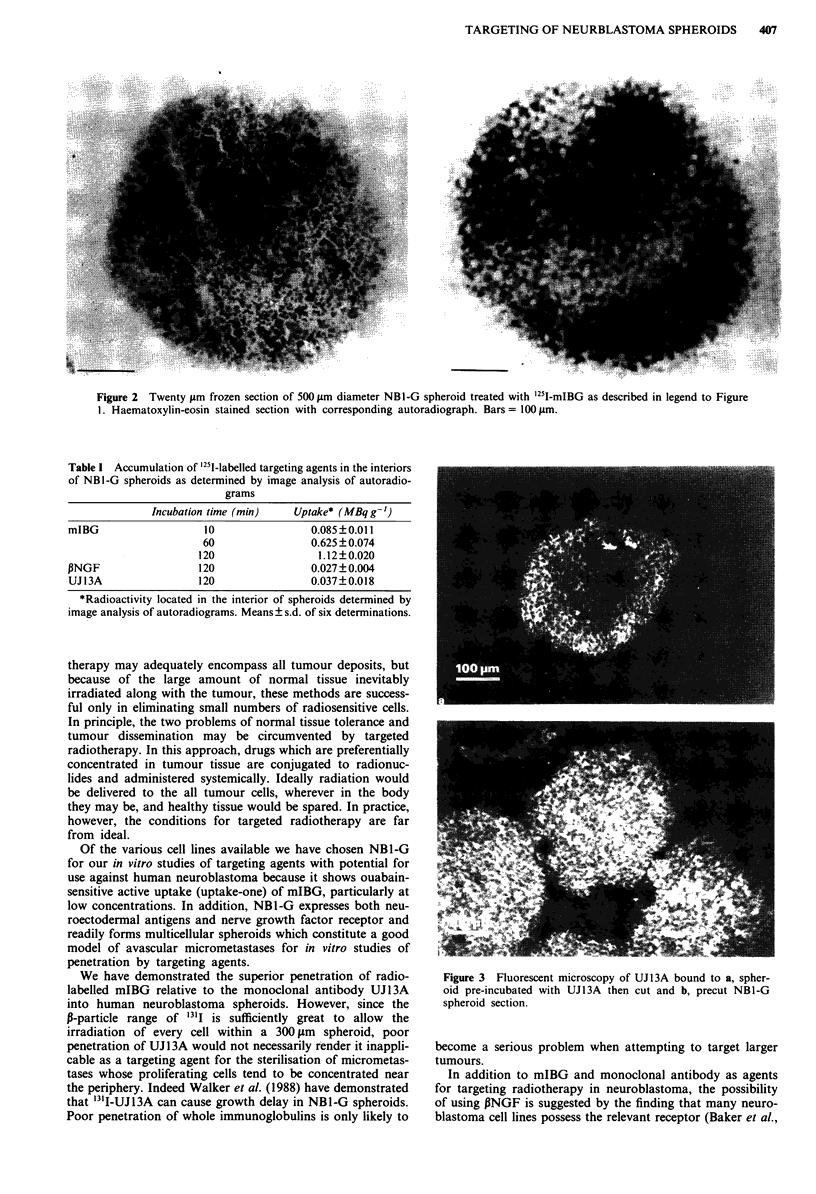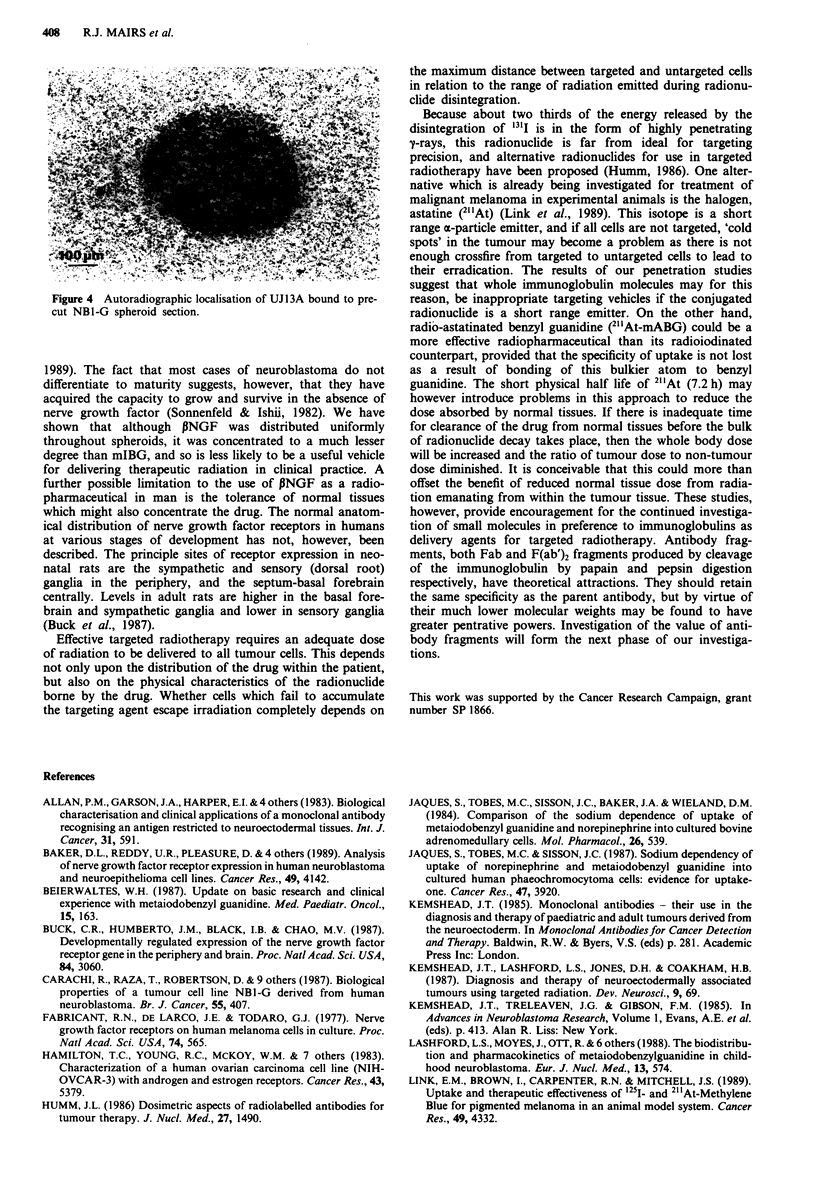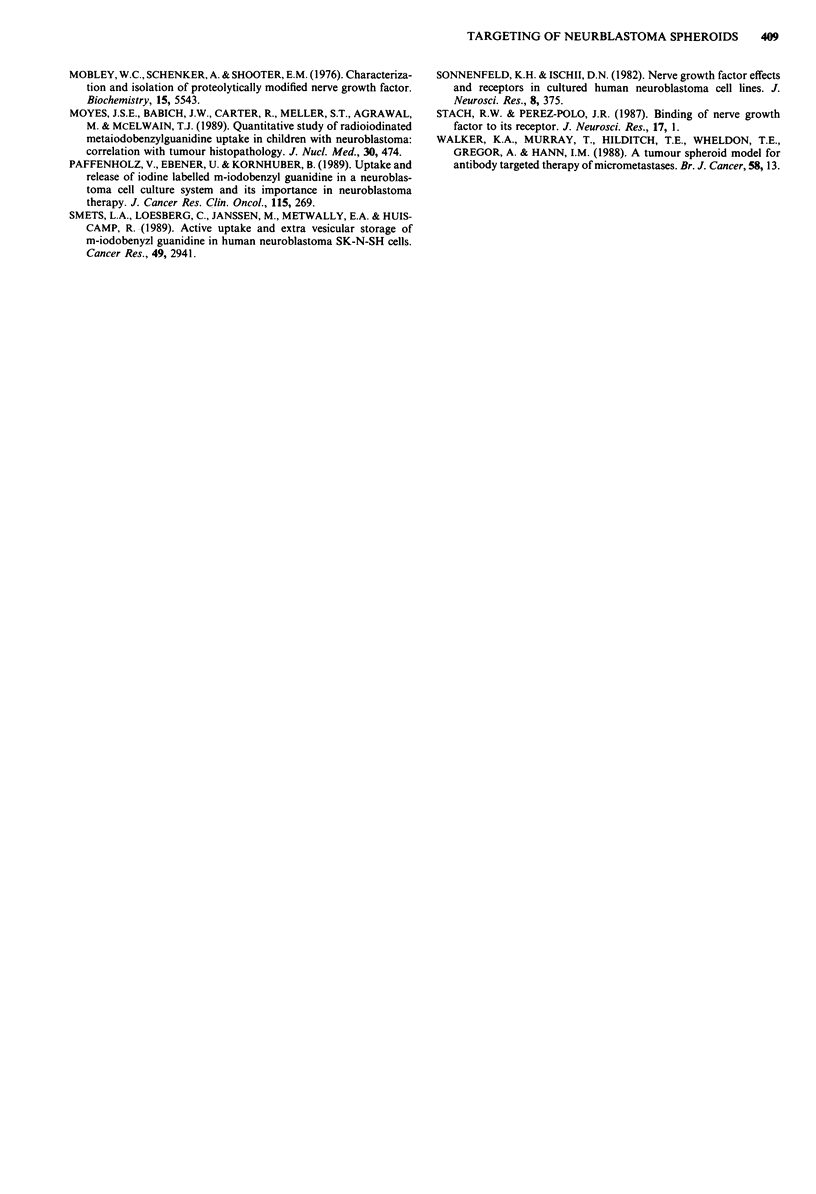# The distribution of alternative agents for targeted radiotherapy within human neuroblastoma spheroids.

**DOI:** 10.1038/bjc.1991.93

**Published:** 1991-03

**Authors:** R. J. Mairs, W. Angerson, M. N. Gaze, T. Murray, J. W. Babich, R. Reid, C. McSharry

**Affiliations:** Department of Radiation Oncology, CRC Beatson Laboratories, Glasgow, UK.

## Abstract

**Images:**


Br. J. Cancer (1991), 63, 404-409 © Macmillan Press Ltd., 1991

### The distribution of alternative agents for targeted radiotherapy within human neuroblastoma spheroids

R.J. Mairs[1], W. Angerson[2], M.N. Gaze[1], T. Murray[3], J.W. Babich[4], R. Reid[5] & C. McSharry[6]

[1]*Department of Radiation Oncology, CRC Beatson Laboratories, Alexander Stone Building, Garscube Estate, Glasgow G61 1BD;* [2]*Department of Surgery, Royal Infirmary, Glasgow G4 0SF; Departments of* [3]*Nuclear Medicine,* [5]*Pathology and* [6]*Bacteriology and Immunology, Western Infirmary, Glasgow G11 6NT;* [4]*Royal Marsden Hospital, Sutton, Surrey SM2 5PT; UK.*

**Summary** This study aims to select the radiopharmaceutical vehicle for targeted radiotherapy of neuroblastoma which is most likely to penetrate readily the centre of micrometastases *in vivo*. The human neuroblastoma cell line NB1-G, grown as multicellular spheroids, provided an *in vitro* model for micrometastases. The radiopharmaceuticals studied were the catecholamine analogue metaiodobenzyl guanidine (mIBG), a specific neuroectodermal monoclonal antibody (UJ13A) and $\beta$ nerve growth factor ($\beta$NGF). Following incubation of each drug with neuroblastoma spheroids, autoradiographs of frozen sections were prepared to demonstrate their relative distributions. mIBG and $\beta$NGF were found to penetrate the centre of spheroids readily although the concentration of mIBG greatly exceeded that of $\beta$NGF. In contrast, UJ13A was only bound peripherally. We conclude that mIBG is the best available vehicle for targeted radiotherapy of neuroblastoma cells with active uptake mechanisms for catecholamines. It is suggested that radionuclides with a shorter range of emissions than $^{131}$I may be conjugated to benzyl guanidine to constitute more effective targeting agents with potentially less toxicity to adjacent normal tissues.

Neuroblastoma is a solid malignant tumour of childhood. Although it is relatively radiosensitive, local radiotherapy alone for neuroblastoma is often inadequate because of the propensity of the tumour for early systemic spread. Some neuroblastoma cells have biochemical peculiarities which enable preferential concentration of catecholamine precursors; they may also express antigens against which monoclonal antibodies have been prepared. Neuroblastoma is therefore regarded as one of the best candidates for targeted radiotherapy (Lashford *et al.*, 1988). It is possible that this form of treatment will be more effective in the eradication of small tumour deposits rather than bulky disease (Kemshead *et al.*, 1987).

Since neuroblastoma arises from undifferentiated sympathetic nerve cells, these tumours are often able to accumulate and store catecholamines more readily than other tissues. This property has lead to the use of the radiopharmaceutical compound metaiodobenzyl guanidine (mIBG), an analogue of the adrenergic neurone blocking drug guanethidine, for the detection, staging and treatment of neuroblastoma (Beierwaltes, 1987). Selective absorption of this drug is thought to be achieved mainly by an active transport process (Jaques *et al.*, 1984) which is sensitive to desmethylimipramine and ouabain (Jaques *et al.*, 1987).

The potential of monoclonal antibodies to target tumours continues to be the focus of much research. One such antibody, UJ13A, binds specifically to neuroectodermal antigens (Allan *et al.*, 1983; Kemshead, 1985). It has affinity for human neuroblastoma and, when conjugated with $^{131}$I, has been shown to retard the growth of neuroblastoma spheroids (Walker *et al.*, 1988). However attempts to treat patients with neuroblastoma by intravenous administration of $^{131}$I-labelled UJ13A have been hampered by slow clearance of the antibody from blood resulting in poor concentration of radioactivity in tumour sites (Kemshead *et al.*, 1985).

A third possible way in which radionuclides can be targeted into neuroblastoma utilises their expression of nerve growth factor receptor which is widely found in neuroblastoma cell lines (Sonnenfeld & Ishii, 1982). The level of expression in tumours is often very much higher than in the corresponding normal cell (Fabricant *et al.*, 1977).

Correspondence: R.J. Mairs.
Received 31 May 1990; and in revised form 24 October 1990.

An increase in the therapeutic potential of targeting antibodies or drugs should be achievable by the replacement of long range $\beta$-emitters such as $^{131}$I with short range $\alpha$-emitters, such as the highly radiotoxic halogen astatine ($^{211}$At) (Humm, 1986). However the effectiveness of agents labelled in this way will be dependent upon their penetration into tumours. We have therefore compared the ability of radioiodinated mIBG, UJ13A and $\beta$NGF to penetrate into multicellular tumour spheroids of a neuroblastoma cell line.

### Materials and methods

### *Cell lines*

The human neuroblastoma cell line used as a model of micrometastases in this study was NB1-G. The biological properties of this line, recently established from a child with stage IV neuroblastoma, have been reported by Carachi *et al.* (1987). We have subsequently demonstrated that this cell line exhibits temperature, sodium and energy dependent active uptake (uptake-one) of mIBG, which can be blocked by ouabain, desmethylimipramine and excess norepinephrine, similar to that seen in adrenal medullary cells (Jaques *et al.*, 1984), phaeochromocytoma cells (Jaques *et al.*, 1987) and in other neuroblastoma cells lines (Smets *et al.*, 1989; Paffenholz *et al.*, 1989). In addition, we have demonstrated that this line also shows binding of radiolabelled $\beta$NGF, which is competitively blocked by excess of unlabelled $\beta$NGF, but not by similar quantities of cytochrome C, a molecule of similar size and charge. This indicates that binding of $\beta$NGF to NB1-G cells is a specific process mediated by nerve growth factor receptors (Stach & Perez-Polo, 1987).

Another cell line, A2780, was used as a negative control because it was not of neural crest origin. This line, kindly supplied by Dr R.F. Ozols of the National Cancer Institute, Bethesda, Maryland, was derived from human ovarian carcinoma, and is a variant of the cell line NIH:OVCAR-3 (Hamilton *et al.*, 1983).

### *Culture conditions*

All cells were grown at 37°C in 5% $CO_2$ in Eagle's Minimal Essential Medium containing 25 mM Hepes buffer, 15% foetal calf serum, 2 mM glutamine, penicillin/streptomycin (100 IU ml$^{-1}$) and amphotericin B (2.5 $\mu$g ml$^{-1}$). All media and supplements were obtained from Gibco (Paisley, UK).

*Reagents*

$^{131}$I-mIBG (specific activity 37–185 MBq mg$^{-1}$ or >1110 MBq mg$^{-1}$) was obtained from Amersham International (Amersham, UK). $^{125}$I-mIBG was prepared as described by Moyes *et al.* (1989), from cold mIBG (IK4 kit, CIS, France) using solid phase ion exchange. The radiochemical purity was greater than 95%, as determined by thin layer chromatography and high performance liquid chromatography.

The neuroectodermal antigen-specific monoclonal antibody, UJ13A, was kindly supplied by Dr J.T. Kemshead, ICRF Laboratories, Frenchay Hospital, Bristol. It was labelled by the iodogen method using carrier free Na($^{125}$I) (Amersham International plc). Following the incubation of 100 μg protein with $^{125}$I, the efficiency of binding of radioiodine was shown by thin layer chromatography to be greater than 80%.

The β-subunit of nerve growth factor (βNGF) was isolated from male mouse submaxillary salivary glands according to the method of Mobley *et al.* (1976) and was further purified by column chromatography. Electrophoresis and HPLC were used to ensure that the βNGF was pure. Radiolabelling was performed as with UJ13A. Thin layer chromatography demonstrated the efficiency of binding to be in excess of 70%.

*Determination of penetrability*

Multicellular tumour spheroids were prepared by continuous stirring of NB1-G cells (2 × 10$^4$ cells ml$^{-1}$) in Techne (Cambridge, UK) stirrer flasks for 3–7 days at 37°C, 5% $CO_2$. NB1-G spheroids with a diameter greater than 300 μm were incubated at 37°C with gentle mixing (Multimix Roller, Luckham Ltd) for 10, 60 or 120 min in culture medium containing 0.14 MBq ml$^{-1}$ of $^{125}$I-mIBG (specific activity 41.7 MBq mg$^{-1}$), $^{125}$I-UJ13A (specific activity 21.7 MBq 100 μg$^{-1}$) or $^{125}$I-βNGF (specific activity 18.7 MBq 100 μg$^{-1}$). They were then washed three times in culture medium, embedded in mounting medium on cutting blocks and frozen by cooling to −30°C. The time between the end of the incubation period and freezing was 60 min. Twenty μm sections were cut in a Bright OTF/AS cryostat at −30°C, transferred to microscope slides and dried on a hot plate at 60°C. The sections were then sealed in cassettes in contact with Kodak PE205 X-ray film for 4 to 14 days before developing. Autoradiographic standards of similar thickness and containing known concentrations of $^{125}$I (Amersham International) were exposed to the same film. Quantitation of isotopic concentration was achieved by measurement of the optical density of autoradiograms using an image analyser (MCID, Imaging Research Inc). Sections were stained with haemotoxylin and eosin for comparison with the autoradiograms.

In addition to autoradiography, fluorescent microscopy was also used to assess the penetration of UJ13A into spheroids. For this, similarly preincubated spheroids were washed, frozen and sectioned as described above, and incubated for 2 h with the fluorescein isothiocyanate-conjugated anti-mouse IgG (whole molecule) antibody (Sigma, Poole, UK). After thorough rinsing with phosphate buffered saline to remove any unbound traces of the second antibody, sections were examined under UV illumination to show distribution of the antibody.

*Controls*

To investigate whether antigen expression was uniform throughout NB1-G spheroids, precut spheroids frozen sections sections were incubated with $^{125}$I-labelled-UJ13A (0.14 MBq ml$^{-1}$) for 2 h. After being thoroughly washed to remove unbound antibody, sections were autoradiographed as described previously. Further precut sections were incubated with non-radiolabelled UJ13A, and the distribution of antibody (representing the sites of antigen expression) examined both by fluorescent microscopy as above and also by an immunoperoxidase staining technique. The latter was performed on sections of both frozen and formalin fixed, paraffin embedded sections. For frozen section, spheroids were embedded in Cryomatrix mounting medium (Shandon Southern Products Ltd) and frozen with compressed $CO_2$ Sections, 7 μm thick, were cut on a cryostat at −24°C and fixed for 10 min in acetone. For paraffin sections, spheroids were fixed in 10% neutral buffered formalin and embedded in paraffin wax using standard techniques. Sections, 2 μm thick, were cut, mounted on poly-L-lysine coated slides and were dewaxed in xylene. For immunostaining, sections were incubated overnight with UJ13A (at a dilution of 1:20 for paraffin sections, and 1:120 for frozen sections), and stained using a biotin/ avidin/peroxidase technique (Vectasain ABC Kit, Vector Laboratories, Burlinghame, CA).

To ensure that any observed binding of antibody was specific for the NB1-G cell line, experiments were repeated with the ovarian carcinoma cell line, A2780.

### Results

Histological examination of haematoxylin and esoin stained NB1-G small spheroid sections revealed an even distribution of mitotic centres. No confluent necrosis was apparent in spheroids of diameter less than 400 μm, although there was evidence of necrosis of single cells which showed nuclear pyknosis (Figure 1). Larger spheroids, however, exhibited central necrotic regions with a peripheral rim of viable cells approximately 200 μm thick (Figure 2).

Incubations of NB1-G spheroids of 300–400 μm diameter with $^{125}$I-labelled targeting agents and subsequent autoradiography of 20 μm thick spheroid sections revealed uniform distribution of mIBG and βNGF whereas UJ13A localisation occurred predominantly on the surface of the spheroids (Figure 1). Subviable regions in larger spheroids (of diameter >500 μm) were associated with reduced uptake of mIBG (Figure 2).

The mean (±s.d.) concentrations of $^{125}$I-mIBG were 0.09 (0.01), 0.63 (0.07) and 1.12 (0.20) MBq g$^{-1}$ after 10, 60 and 120 min incubation respectively. $^{125}$I-βNGF was also uniformly distributed, but at a much lower concentration (2–3% of that of mIBG). $^{125}$I-UJ13A was bound predominantly in a thin layer around the periphery of spheroids, with little penetration to the interior (3–4% relative to mIBG) – see Table I. Prolonged incubation (24 h) of spheroids with βNGF did not result in uptake levels greater than that seen at 2 h. Binding of antibody only to the periphery was also observed by fluorescent microscopy of spheroids sectioned after incubation with UJ13A (Figure 3a).

In order to determine whether peripheral antibody binding to spheroids was a consequence of differential antigen expression between surface cells and those located in the interior, spheroids of various diameters (100–600 μm) were sectioned and exposed to $^{125}$I-UJ13A for 2 h. Autoradiographs showed almost uniform binding of antibody throughout the cross-section (Figure 4). Similarly, fluorescent microscopy (Figure 3b) and immunoperoxidase staining of precut sections incubated with unlabelled antibody both revealed binding of UJ13A throughout the sections, whereas binding to the A2780 control spheroids only occurred non-specifically in the necrotic regions. Therefore the inefficient concentration of UJ13A in the deeper portions of NB1-G spheroids appears to be due to an inability of the monoclonal antibody to penetrate beyond the outer cell layers.

### Discussion

The principal limitation of radiotherapy as a curative modality is the limited radiation tolerance of normal tissues. If a tumour is clearly circumscribed it is often possible to deliver a curative dose of radiation without causing critical damage to adjacent normal tissues. However many tumours have a propensity for early systemic spread, making curative local irradiation impossible. Wide field and whole body radio-

**Figure 1** Twenty μm frozen sections of 300–400 μm diameter NB1-G spheroids treated for 2 h with 0.14 MBq $ml^{-1}$ **a**, $^{125}$I-mIBG (specific activity 41.7 MBq $mg^{-1}$), **b**, $^{125}$I-UJ13A (specific activity 21.7 MBq 100 $\mu g^{-1}$ protein) or **c**, $^{125}$I-βNGF (specific activity 18.7 MBq 100 $\mu g^{-1}$). Haematoxylin-eosin stained sections with corresponding autoradiographs. Bars = 100 μm.

**Figure 2** Twenty μm frozen section of 500 μm diameter NB1-G spheroid treated with $^{125}$I-mIBG as described in legend to Figure 1. Haematoxylin-eosin stained section with corresponding autoradiograph. Bars = 100 μm.

**Table I** Accumulation of $^{125}$I-labelled targeting agents in the interiors of NB1-G spheroids as determined by image analysis of autoradiograms

| | *Incubation time (min)* | *Uptake* (MBq g$^{-1}$)* |
|---|---|---|
| mIBG | 10 | 0.085±0.011 |
| | 60 | 0.625±0.074 |
| | 120 | 1.12±0.020 |
| βNGF | 120 | 0.027±0.004 |
| UJ13A | 120 | 0.037±0.018 |

*Radioactivity located in the interior of spheroids determined by image analysis of autoradiograms. Means ± s.d. of six determinations.

therapy may adequately encompass all tumour deposits, but because of the large amount of normal tissue inevitably irradiated along with the tumour, these methods are successful only in eliminating small numbers of radiosensitive cells. In principle, the two problems of normal tissue tolerance and tumour dissemination may be circumvented by targeted radiotherapy. In this approach, drugs which are preferentially concentrated in tumour tissue are conjugated to radionuclides and administered systemically. Ideally radiation would be delivered to the all tumour cells, wherever in the body they may be, and healthy tissue would be spared. In practice, however, the conditions for targeted radiotherapy are far from ideal.

Of the various cell lines available we have chosen NB1-G for our *in vitro* studies of targeting agents with potential for use against human neuroblastoma because it shows ouabain-sensitive active uptake (uptake-one) of mIBG, particularly at low concentrations. In addition, NB1-G expresses both neuroectodermal antigens and nerve growth factor receptor and readily forms multicellular spheroids which constitute a good model of avascular micrometastases for *in vitro* studies of penetration by targeting agents.

We have demonstrated the superior penetration of radiolabelled mIBG relative to the monoclonal antibody UJ13A into human neuroblastoma spheroids. However, since the β-particle range of $^{131}$I is sufficiently great to allow the irradiation of every cell within a 300 μm spheroid, poor penetration of UJ13A would not necessarily render it inapplicable as a targeting agent for the sterilisation of micrometastases whose proliferating cells tend to be concentrated near the periphery. Indeed Walker *et al.* (1988) have demonstrated that $^{131}$I-UJ13A can cause growth delay in NB1-G spheroids. Poor penetration of whole immunoglobulins is only likely to

**Figure 3** Fluorescent microscopy of UJ13A bound to **a**, spheroid pre-incubated with UJ13A then cut and **b**, precut NB1-G spheroid section.

become a serious problem when attempting to target larger tumours.

In addition to mIBG and monoclonal antibody as agents for targeting radiotherapy in neuroblastoma, the possibility of using βNGF is suggested by the finding that many neuroblastoma cell lines possess the relevant receptor (Baker *et al.*,

**Figure 4** Autoradiographic localisation of UJ13A bound to precut NB1-G spheroid section.

1989). The fact that most cases of neuroblastoma do not differentiate to maturity suggests, however, that they have acquired the capacity to grow and survive in the absence of nerve growth factor (Sonnenfeld & Ishii, 1982). We have shown that although βNGF was distributed uniformly throughout spheroids, it was concentrated to a much lesser degree than mIBG, and so is less likely to be a useful vehicle for delivering therapeutic radiation in clinical practice. A further possible limitation to the use of βNGF as a radiopharmaceutical in man is the tolerance of normal tissues which might also concentrate the drug. The normal anatomical distribution of nerve growth factor receptors in humans at various stages of development has not, however, been described. The principle sites of receptor expression in neonatal rats are the sympathetic and sensory (dorsal root) ganglia in the periphery, and the septum-basal forebrain centrally. Levels in adult rats are higher in the basal forebrain and sympathetic ganglia and lower in sensory ganglia (Buck et al., 1987).

Effective targeted radiotherapy requires an adequate dose of radiation to be delivered to all tumour cells. This depends not only upon the distribution of the drug within the patient, but also on the physical characteristics of the radionuclide borne by the drug. Whether cells which fail to accumulate the targeting agent escape irradiation completely depends on the maximum distance between targeted and untargeted cells in relation to the range of radiation emitted during radionuclide disintegration.

Because about two thirds of the energy released by the disintegration of $^{131}I$ is in the form of highly penetrating γ-rays, this radionuclide is far from ideal for targeting precision, and alternative radionuclides for use in targeted radiotherapy have been proposed (Humm, 1986). One alternative which is already being investigated for treatment of malignant melanoma in experimental animals is the halogen, astatine ($^{211}At$) (Link et al., 1989). This isotope is a short range α-particle emitter, and if all cells are not targeted, 'cold spots' in the tumour may become a problem as there is not enough crossfire from targeted to untargeted cells to lead to their erradication. The results of our penetration studies suggest that whole immunoglobulin molecules may for this reason, be inappropriate targeting vehicles if the conjugated radionuclide is a short range emitter. On the other hand, radio-astatinated benzyl guanidine ($^{211}At$-mABG) could be a more effective radiopharmaceutical than its radioiodinated counterpart, provided that the specificity of uptake is not lost as a result of bonding of this bulkier atom to benzyl guanidine. The short physical half life of $^{211}At$ (7.2 h) may however introduce problems in this approach to reduce the dose absorbed by normal tissues. If there is inadequate time for clearance of the drug from normal tissues before the bulk of radionuclide decay takes place, then the whole body dose will be increased and the ratio of tumour dose to non-tumour dose diminished. It is conceivable that this could more than offset the benefit of reduced normal tissue dose from radiation emanating from within the tumour tissue. These studies, however, provide encouragement for the continued investigation of small molecules in preference to immunoglobulins as delivery agents for targeted radiotherapy. Antibody fragments, both Fab and $F(ab')_2$ fragments produced by cleavage of the immunoglobulin by papain and pepsin digestion respectively, have theoretical attractions. They should retain the same specificity as the parent antibody, but by virtue of their much lower molecular weights may be found to have greater pentrative powers. Investigation of the value of antibody fragments will form the next phase of our investigations.

This work was supported by the Cancer Research Campaign, grant number SP 1866.

### References

ALLAN, P.M., GARSON, J.A., HARPER, E.I. & 4 others (1983). Biological characterisation and clinical applications of a monoclonal antibody recognising an antigen restricted to neuroectodermal tissues. *Int. J. Cancer*, **31**, 591.

BAKER, D.L., REDDY, U.R., PLEASURE, D. & 4 others (1989). Analysis of nerve growth factor receptor expression in human neuroblastoma and neuroepithelioma cell lines. *Cancer Res.*, **49**, 4142.

BEIERWALTES, W.H. (1987). Update on basic research and clinical experience with metaiodobenzyl guanidine. *Med. Paediatr. Oncol.*, **15**, 163.

BUCK, C.R., HUMBERTO, J.M., BLACK, I.B. & CHAO, M.V. (1987). Developmentally regulated expression of the nerve growth factor receptor gene in the periphery and brain. *Proc. Natl Acad. Sci. USA*, **84**, 3060.

CARACHI, R., RAZA, T., ROBERTSON, D. & 9 others (1987). Biological properties of a tumour cell line NB1-G derived from human neuroblastoma. *Br. J. Cancer*, **55**, 407.

FABRICANT, R.N., DE LARCO, J.E. & TODARO, G.J. (1977). Nerve growth factor receptors on human melanoma cells in culture. *Proc. Natl Acad. Sci. USA*, **74**, 565.

HAMILTON, T.C., YOUNG, R.C., McKOY, W.M. & 7 others (1983). Characterization of a human ovarian carcinoma cell line (NIH-OVCAR-3) with androgen and estrogen receptors. *Cancer Res.*, **43**, 5379.

HUMM, J.L. (1986) Dosimetric aspects of radiolabelled antibodies for tumour therapy. *J. Nucl. Med.*, **27**, 1490.

JAQUES, S., TOBES, M.C., SISSON, J.C., BAKER, J.A. & WIELAND, D.M. (1984). Comparison of the sodium dependence of uptake of metaiodobenzyl guanidine and norepinephrine into cultured bovine adrenomedullary cells. *Mol. Pharmacol.*, **26**, 539.

JAQUES, S., TOBES, M.C. & SISSON, J.C. (1987). Sodium dependency of uptake of norepinephrine and metaiodobenzyl guanidine into cultured human phaeochromocytoma cells: evidence for uptake-one. *Cancer Res.*, **47**, 3920.

KEMSHEAD, J.T. (1985). Monoclonal antibodies – their use in the diagnosis and therapy of paediatric and adult tumours derived from the neuroectoderm. In *Monoclonal Antibodies for Cancer Detection and Therapy*. Baldwin, R.W. & Byers, V.S. (eds) p. 281. Academic Press Inc: London.

KEMSHEAD, J.T., LASHFORD, L.S., JONES, D.H. & COAKHAM, H.B. (1987). Diagnosis and therapy of neuroectodermally associated tumours using targeted radiation. *Dev. Neurosci.*, **9**, 69.

KEMSHEAD, J.T., TRELEAVEN, J.G. & GIBSON, F.M. (1985). In *Advances in Neuroblastoma Research*, Volume 1, Evans, A.E. *et al.* (eds). p. 413. Alan R. Liss: New York.

LASHFORD, L.S., MOYES, J., OTT, R. & 6 others (1988). The biodistribution and pharmacokinetics of metaiodobenzylguanidine in childhood neuroblastoma. *Eur. J. Nucl. Med.*, **13**, 574.

LINK, E.M., BROWN, I., CARPENTER, R.N. & MITCHELL, J.S. (1989). Uptake and therapeutic effectiveness of $^{125}I$- and $^{211}At$-Methylene Blue for pigmented melanoma in an animal model system. *Cancer Res.*, **49**, 4332.

MOBLEY, W.C., SCHENKER, A. & SHOOTER, E.M. (1976). Characterization and isolation of proteolytically modified nerve growth factor. *Biochemistry*, **15,** 5543.

MOYES, J.S.E., BABICH, J.W., CARTER, R., MELLER, S.T., AGRAWAL, M. & MCELWAIN, T.J. (1989). Quantitative study of radioiodinated metaiodobenzylguanidine uptake in children with neuroblastoma: correlation with tumour histopathology. *J. Nucl. Med.*, **30,** 474.

PAFFENHOLZ, V., EBENER, U. & KORNHUBER, B. (1989). Uptake and release of iodine labelled m-iodobenzyl guanidine in a neuroblastoma cell culture system and its importance in neuroblastoma therapy. *J. Cancer Res. Clin. Oncol.*, **115,** 269.

SMETS, L.A., LOESBERG, C., JANSSEN, M., METWALLY, E.A. & HUISCAMP, R. (1989). Active uptake and extra vesicular storage of m-iodobenyzl guanidine in human neuroblastoma SK-N-SH cells. *Cancer Res.*, **49,** 2941.

SONNENFELD, K.H. & ISCHII, D.N. (1982). Nerve growth factor effects and receptors in cultured human neuroblastoma cell lines. *J. Neurosci. Res.*, **8,** 375.

STACH, R.W. & PEREZ-POLO, J.R. (1987). Binding of nerve growth factor to its receptor. *J. Neurosci. Res.*, **17,** 1.

WALKER, K.A., MURRAY, T., HILDITCH, T.E., WHELDON, T.E., GREGOR, A. & HANN, I.M. (1988). A tumour spheroid model for antibody targeted therapy of micrometastases. *Br. J. Cancer*, **58,** 13.